# Expression of Concern: Progesterone receptor antagonist provides palliative effects for uterine leiomyoma through a Bcl-2/Beclin1-dependent mechanism

**DOI:** 10.1042/BSR-20190094_EOC

**Published:** 2021-04-06

**Keywords:** apoptosis, autophagy, mifepristone, progesterone receptor antagonist, progesterone receptor-M, uterine leiomyoma

The Editorial Office has been made aware of potential issues surrounding the scientific validity of this paper, hence has issued an expression of concern to notify readers whilst the Editorial Office investigates.

